# Diabetes mellitus, cardiovascular and chronic respiratory diseases in Germany and Europe – results of the European Health Interview Survey (EHIS 3, 2018 – 2020)

**DOI:** 10.25646/12920

**Published:** 2024-12-04

**Authors:** Jens Baumert, Giselle Sarganas, Ronny Kuhnert, Roma Thamm, Henriette Steppuhn, Julia Waldhauer, Jens Hoebel, Hannelore Neuhauser, Christin Heidemann

**Affiliations:** Robert Koch Institute, Department of Epidemiology and Health Monitoring, Berlin, Germany

**Keywords:** Diabetes mellitus, Cardiovascular diseases, Chronic respiratory diseases, Chronic diseases, Self-assessed health, Limitations, Education, Europe

## Abstract

**Background:**

In Europe, the health situation is primarily influenced by non-communicable diseases. Comparable information on key indicators for the European region can highlight potential areas for improvement in prevention and care.

**Method:**

Based on EHIS 3, age-standardised prevalences of three disease groups and two indicators of self-assessed health among those affected were presented for Germany (*n* = 22,708) and the average of 29 European countries (*n* = 301,960).

**Results:**

The disease prevalence estimates in Germany were higher compared to the European average: diabetes 8.4 % vs. 7.4 %, cardiovascular diseases 6.8 % vs. 5.7 %, chronic respiratory diseases 11.4 % vs. 7.9 %. Likewise, the proportion with self-assessed very good or good general health among those affected was also higher in Germany (diabetes 35.8 % vs. 30.7 %, cardiovascular diseases 25.3 % vs. 18.9 %, chronic respiratory diseases 44.7 % vs. 41.9 %). For limitations in everyday activities, higher proportions were found in Germany for diabetes (65.6 % vs. 60.6 %) and chronic respiratory diseases (64.5 % vs. 57.6 %). Germany showed similar gender-, age- and education-specific differences for disease prevalence, but in part less pronounced differences for the indicators of self-assessed health than the European average.

**Conclusions:**

Further analysis of the differences for the indicators considered between Germany and the European average and the possible underlying factors, such as differences in prevention, diagnosis, disease severity and care, is required. The educational inequalities observed across Europe suggest considerable potential for promoting health equity.

## 1. Introduction

Healthcare systems in European countries are facing similar challenges such as demographic change, emerging health threats (e.g. pandemics) or socio-economic inequalities in health and care [1]. In order to respond appropriately and develop strategies to improve health, regular and comprehensive information on key health indicators is required [1]. The European Health Interview Survey (EHIS), which is conducted approximately every five years and is now mandatory for all Member States of the European Union (EU), was launched at the end of the 2000s for the areas of health status, determinants and care [2].

The incidence of disease and mortality in European countries is dominated by non-communicable diseases. These manifest themselves mainly in middle and old age, with the result that the prevalence of disease has increased in recent decades favored by the demographic change and the increase in risk factors [3, 4], e.g. an increase in the prevalence of cardiovascular disease in the period 1990 – 2019 from 12.6 % to 13.8 % in the population aged 20 and over in Europe [5]. Placing the prevalence of non-communicable diseases in Germany in the European context can help to identify conspicuous features and derive a possible need for improvements in prevention and care.

In terms of disease frequency and mortality, diabetes mellitus, cardiovascular diseases (especially coronary heart disease and stroke), cancer and chronic respiratory diseases (especially chronic obstructive pulmonary disease (COPD) and bronchial asthma) are among the most important non-communicable diseases [6]. Those affected usually require lifelong care (e.g. medication, check-ups, rehabilitation). An often high degree of self-management required and possible consequences of the disease can also have an influence on the subjective health assessment of those affected, so that they rate their general state of health worse than those not affected and report more frequently on health-related limitations in everyday activities [6]. These two indicators of self-assessed health are among the three key indicators of health status as established components of the ‘Minimum European Health Module’ (MEHM) [7] and are recommended by the European Commission for monitoring the health status of a population [8].

This article contains an analysis of the prevalence of the three disease groups diabetes mellitus, cardiovascular diseases and chronic respiratory diseases in adults (aged 18 and over) in Germany compared to the European average from 29 countries. The analyses are based on the European Health Interview Survey EHIS Wave 3 conducted from 2018 to 2020 [9]. In the presence of diabetes, cardiovascular disease and chronic respiratory diseases, the proportions of people with self-assessed very good or good general health and with self-assessed severe or moderate health-related limitations in everyday activities are also examined.

## 2. Methods

### 2.1 Sample design and study implementation

The aim of the EHIS survey is to regularly provide comparable health data from the EU Member States and thus enable observations to be made in the development of health indicators in Europe [10]. The target population is the population living in private households with permanent residence in the respective country aged 15 years or older. EHIS 3 was conducted from 2018 to 2020 in all EU Member States as well as in Iceland, Norway, Albania, Serbia and Turkey. A quality report contains detailed information on the methodological approach of the individual countries [11]. The aggregated data can be found on the website of the EU statistical office (Eurostat) [12]. Albania, France, Turkey and the United Kingdom have not yet made any data publicly available [13]. For research purposes, anonymized data at participant level (microdata) for the EU Member States can be requested from Eurostat [14]. The data set used for the present analyses contains data from 29 European countries (Austria, Belgium, Bulgaria, Croatia, Cyprus, the Czech Republic, Denmark, Estonia, Finland, Germany, Greece, Hungary, Iceland, Ireland, Italy, Latvia, Lithuania, Luxembourg, Malta, the Netherlands, Norway, Poland, Portugal, Romania, Serbia, Slovakia, Slovenia, Spain and Sweden), of which 26 are EU Member States and three are non-EU countries (Iceland, Norway, Serbia).

**Key statements**

► Compared to the European average, Germany showed higher prevalence estimates for the disease groups diabetes, cardiovascular and chronic respiratory diseases.
► The prevalence estimates observed in Germany and on average in Europe increased with age for both sexes.
► The highest prevalence estimates in Germany and the European average were found in the low education group.
► In Germany, adults with diabetes, cardiovascular or chronic respiratory diseases rated their health better than the European average.
► In Germany, health-related limitations in everyday activities were more common in diabetes and chronic respiratory diseases than for the European average.

The analyses reported here are based on data from 301,960 participants (162,395 women, 139,565 men) aged 18 and over who answered the EHIS 3 survey themselves or through a relative, including 22,708 participants from Germany (11,968 women, 10,740 men). For Malta and Iceland, data is available from the age of 20 years.

### 2.2 Indicators

The regulation on the implementation of the EHIS specified the survey content to be collected, including the response options and the codes to be transmitted to Eurostat. In addition, the wording of the questions and their response categories as well as the order in which they are asked were explained in a methodological manual and made available in the form of a sample questionnaire (in English) [9]. Compliance with the rules and recommendations designed as guidelines was essential for ensuring harmonized and high-quality health data in the EU.

#### Diabetes mellitus, cardiovascular and chronic respiratory diseases

The data on the 12-month prevalence of the selected disease groups is based on the answers to the following question: ‘This section deals with lasting diseases and chronic health problems. Please do not include temporary health problems. In the past 12 months, have you had any of the following diseases or health problems?’ In a list attached to the questionnaire, respondents were asked about the presence of individual diseases and complaints, for each of which the answers ‘yes’, ‘no’ or ‘don’t know’ could be given. The present study takes into account the information collected on (1) diabetes mellitus (queried as ‘diabetes, not gestational diabetes’), (2) cardiovascular diseases: coronary heart disease (queried as ‘heart attack’, ‘chronic consequences of heart attack’ and ‘coronary heart disease or angina pectoris’) and stroke (queried as ‘stroke’ and ‘chronic consequences of stroke’) and (3) chronic respiratory diseases: chronic obstructive pulmonary disease (queried as ‘chronic bronchitis’, ‘chronic obstructive pulmonary disease, emphysema’) and bronchial asthma (queried as ‘asthma, including allergic asthma’).

#### Self-assessed general health status and health-related limitations in everyday activities

Data on two indicators of self-assessed health status were selected for this analysis as part of the Minimum European Health Module (MEHM) and as a central component of all national health surveys in the EU [1, 7].

The indicator for self-assessed general health is measured with the following question recommended by the World Health Organization (WHO): ‘How is your health in general?’ Respondents were asked to choose one of five possible answers: ‘very good’, ‘good’, ‘fair’, ‘bad’ or ‘very bad’. For the analysis, the answers were grouped into two categories: very good/good vs. fair/bad/very bad.

The indicator for health-related limitations in everyday activities (Global Activity Limitation Indicator, GALI) was surveyed in two stages. The first question was: ‘Are you limited by a health problem in activities of your normal everyday life? Would you say you are…’, with the response options ‘… severely limited’, ‘… moderately limited’ and ‘… not limited’. Respondents who gave one of the first two answer options were asked further: ‘How long have you been limited?’ The possible answers were ‘less than 6 months’ and ‘6 months or longer’. For the analysis, the answers were grouped into two categories: severely/moderately limited for at least six months vs. not limited or severely/moderately limited for less than six months.

#### Sociodemographics

In addition to the gender of the respondents, age was taken into account as a determinant of chronic disease. The following age groups were used: 18 to 44 years, 45 to 64 years, 65 to 74 years and 75 years and older (persons under 18 years were excluded from the analyses). With regard to the non-communicable diseases under consideration, these age groups represent the increase in prevalence in different phases of life with a sufficient number of cases (the highest age group available in the data set is the 75 years and older age group). The educational status of the respondents was classified according to the 2011 version of the International Standard Classification of Education (ISCED) using information on educational and vocational qualifications [15]. In order to be able to make comparisons between individual education groups, the standard classification aggregates ISCED levels 0 to 2 as the low education group, ISCED levels 3 to 4 as the medium education group and ISCED levels 5 to 8 as the high education group and was implemented accordingly for the present analyses [16].

#### 2.3 Statistical analyses

In order to consider each country in proportion to its population size, the analyses were carried out using a weighting factor. In the weighting for the European comparison, the educational status characteristic is not taken into account in accordance with Eurostat recommendations. The household ID was used as cluster variable. In order to compensate for potentially distorting age differences between the countries, a direct age standardisation was carried out in the calculation. The age structures of the country samples were each adjusted to the European standard population for 2013 [17]. The figures for the European average refer to all 29 countries included in the analyses, i.e. including Germany. For each of the indicators considered, the percentage share was calculated with 95 % logit confidence intervals (95 % CI) stratified by gender, age and education. A statistically significant difference between groups was assumed if the corresponding p-value in the Rao-Scott chi-square test was less than 0.05. All analyses were performed using the programs R version R 4.3.0 and SAS version 9.4.

## 3. Results

### 3.1 Diabetes mellitus

#### Prevalence

**Figure 1:**
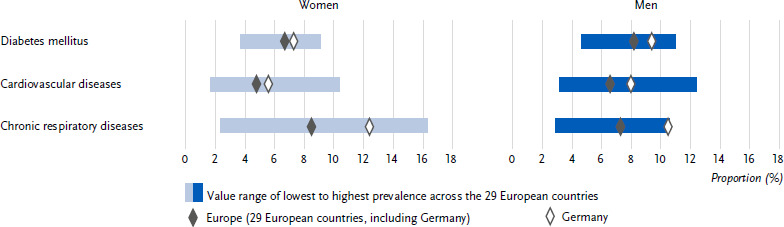
Age-standardised prevalence of diabetes mellitus, cardiovascular and chronic respiratory diseases in the past twelve months in Germany and Europe for women and men (*n* = 162,395 women, *n* = 139,565 men). Source: EHIS wave 3 (2018 – 2020)

**Figure 2:**
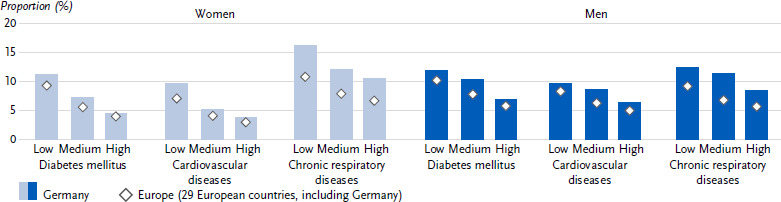
Age-standardised prevalence of diabetes mellitus, cardiovascular and chronic respiratory diseases in the past twelve months in Germany and Europe by education group among women and men (*n* = 162,395 women, *n* = 139,565 men). Source: EHIS wave 3 (2018 – 2020)

Overall, 8.4 % (age-standardised) of all respondents in Germany reported the presence of diabetes in the past twelve months. This proportion was lower for women (7.3 %) than for men (9.4 %) (Figure 1 and Annex Table 1). The prevalence of diabetes increased steadily for both sexes across the age groups (Annex Table 1). The European average 12-month diabetes prevalence with 7.4 % was lower than in Germany. The European average diabetes prevalence also differed between the sexes (women: 6.7 %, men: 8.2 %, Figure 1 and Annex Table 1) and increased with age (Annex Table 1). Both in Germany and in the European average, women and men showed a gradual increase in diabetes prevalence with decreasing education (Figure 2 and Annex Table 1).

#### Self-assessed general health status and health-related limitations in everyday activities

**Figure 3:**
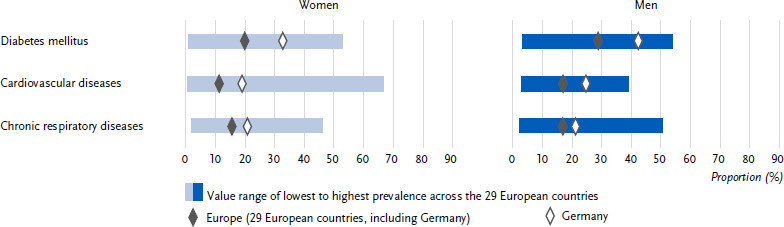
Age-standardised prevalence of self-assessed very good or good health status among people with diabetes mellitus, cardiovascular and chronic respiratory diseases in the past twelve months in Germany and Europe for women and men (*n* = 162,395 women, *n* = 139,565 men). Source: EHIS wave 3 (2018 – 2020)

**Figure 4:**
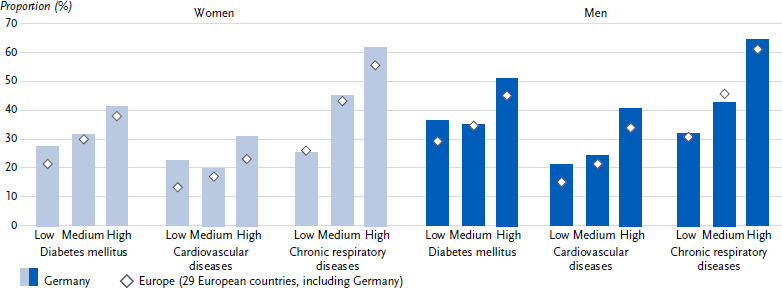
Age-standardised prevalence of self-assessed very good or good health status among people with diabetes mellitus, cardiovascular and chronic respiratory diseases in the past twelve months in Germany and Europe by education group for women and men (*n* = 162,395 women, *n* = 139,565 men). Source: EHIS wave 3 (2018 – 2020)

The prevalence of self-assessed very good or good general health among people with diabetes in the past twelve months was higher in Germany (35.8 %) than the European average of 30.7 % (Annex Table 2). This prevalence was lower in women than in men; this applies both to Germany (31.7 % vs. 38.9 %) and to the European average (26.1 % vs. 34.4 %) (Figure 3 and Annex Table 2). There is no clear trend across the age groups for Germany in terms of the proportion of women and men with self-assessed health as very good or good, whereas this proportion decreased steadily with increasing age in the European average (Annex Table 2). In Germany, women and men with diabetes from the low and middle education groups combined had a lower proportion with self-assessed health as very good or good than those in the high education group, while the European average shows successively decreasing proportions with decreasing education (Figure 4 and Annex Table 2).

**Figure 5:**
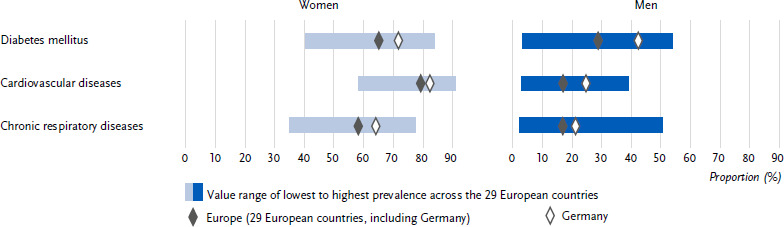
Age-standardised prevalence of self-assessed severe or moderate health-related limitations in everyday activities for at least six months among people with diabetes mellitus, cardiovascular and chronic respiratory diseases in the past twelve months in Germany and Europe for women and men (*n* = 162,395 women, *n* = 139,565 men). Source: EHIS wave 3 (2018 – 2020)

**Figure 6:**
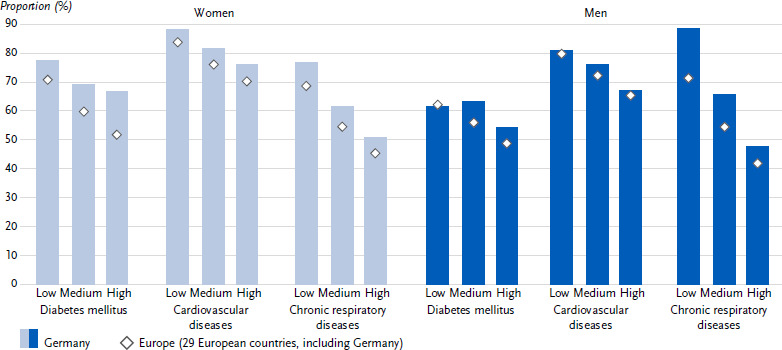
Age-standardised prevalence of self-assessed severe or moderate health-related limitations in everyday activities for at least six months among people with diabetes mellitus, cardiovascular and chronic respiratory diseases in the past twelve months in Germany and Europe by education group for women and men (*n* = 162,395 women, *n* = 139,565 men). Source: EHIS wave 3 (2018 – 2020)

The prevalence of severely or moderately assessed health-related limitations in everyday activities for at least six months in people with diabetes was higher in Germany than the European average (65.6 % vs. 60.6 %, Annex Table 3). This prevalence was higher in women than in men; this was true for both Germany (71.7 % vs. 61.0 %) and the European average (65.1 % vs. 57.0 %) (Figure 5 and Annex Table 3). Across the age groups, there was no clear trend for women and men in Germany; on average in Europe, however, the prevalence increased significantly with age (Annex Table 3). With regard to education, women and men in Germany showed no differences in the prevalence of severe or moderate health-related limitations in everyday activities; the European average, on the other hand, showed an educational gradient with increasing health-related limitations with decreasing education (Figure 6 and Annex Table 3).

### 3.2 Cardiovascular diseases

#### Prevalence

In Germany, 6.8 % (age-standardised) of all respondents reported the presence of coronary heart disease or a stroke or their chronic consequences in the past twelve months. This proportion was lower for women (5.6 %) than for men (8.0 %) (Figure 1 and Annex Table 1). The 12-month prevalence of these cardiovascular diseases increased continuously in both sexes across the age groups (Annex Table 1). In comparison, the prevalence of cardiovascular diseases in the European average was lower than in Germany at 5.7 %. The prevalence of cardiovascular diseases also differed between the sexes in the European average (women: 4.8 %, men: 6.6 %, Figure 1 and Annex Table 1) and increased with age (Annex Table 1). In Germany, as well as in the European average, the combined prevalence was higher among women and men in the low and middle education groups than among people in the high education group (Figure 2 and Annex Table 1).

#### Self-assessed general health status and health-related limitations in everyday activities

The prevalence of self-assessed very good or good health among people with cardiovascular disease in the past twelve months was higher in Germany (25.3 %) than the European average of 18.9 % (Annex Table 2). While the prevalence in Germany did not differ between women and men (21.8 % vs. 27.7 %), the European average prevalence was lower in women than in men (15.6 % vs. 21.2 %) (Figure 3 and Annex Table 2). While there is no clear age trend for the proportion of women and men in Germany with self-assessed very good or good health, the proportion in the European average decreased with increasing age (Annex Table 2). In Germany, the proportion of men, but not women, with a very good or good self-assessment of their health was lower in the low and medium education groups than in the high education group (Figure 4 and Annex Table 2). The European average showed an increase with increasing education for both genders.

The proportion of people with cardiovascular disease with self-assessed severe or moderate health-related limitations in everyday activities for at least six months was similar in Germany compared to the European average (78.1 % vs. 76.3 %) (Annex Table 3). This proportion was higher for women than for men; this was true for both Germany (82.3 % vs. 75.2 %) and the European average (79.2 % vs. 74.2 %) (Figure 5 and Annex Table 3). Across the age groups, the European average showed a clear increase in prevalence among women and men; in Germany, no clear age trend was observed (Annex Table 3). With regard to education, women and men in Germany showed no significant differences in the prevalence of severe to moderate health-related limitations in everyday activities; the European average showed higher proportions for people in the low and medium education groups combined compared to people in the high education group (Figure 6 and Annex Table 3).

### 3.3 Chronic respiratory diseases

#### Prevalence

The proportion of people with chronic respiratory diseases in the past twelve months in Germany was 11.4 % (age-standardized). At 12.4 %, the proportion was higher for women than for men at 10.5 % (Figure 1 and Annex Table 1). Over the course of age, the prevalence in Germany showed an increase between the age groups 18 to 44 years and 45 to 64 years and then largely plateaued (Annex Table 1). The European average prevalence of 7.9 % was lower than in Germany. The prevalence of chronic respiratory diseases also differed between the sexes in the European average (women: 8.5 %, men: 7.3 %, Figure 1 and Annex Table 1) and increased with age (Annex Table 1). Both in Germany and in the European average, women and men in the low and middle education groups combined had higher prevalence estimates than people in the high education group (Figure 2 and Annex Table 1).

#### Self-assessed general health status and health-related limitations in everyday activities

The prevalence of self-assessed very good or good health in people with chronic respiratory diseases in the past twelve months was higher in Germany (44.7 %) than the European average of 41.9 % (Annex Table 2). While the prevalence in Germany did not differ between women and men (43.8 % vs. 45.8 %), the European average prevalence was lower in women than in men (40.6 % vs. 43.4 %) (Figure 3 and Annex Table 2). In Germany and also in the European average, the proportion of women and men with a self-assessed health status of very good or good decreased with increasing age (Annex Table 2) and increased with increasing education (Figure 4 and Annex Table 2).

The prevalence of self-assessed severe or moderate health-related limitations in everyday activities for at least six months in people with chronic respiratory diseases was higher in Germany than the European average (64.5 % vs. 57.6 %) (Annex Table 3). There were no differences between women and men in Germany (64.1 % vs. 64.8 %) and the European average (58.2 % vs. 56.8 %) (Figure 5 and Annex Table 3). Over the course of age, there was an increase in the prevalence of severe or moderate health-related restrictions in everyday activities in women and men with chronic respiratory diseases, both in Germany and the European average (Annex Table 3). In Germany and on average in Europe, a higher proportion of women and men with chronic respiratory diseases who belonged to the low and middle education group had severe to moderate health-related limitations in everyday activities compared to people in the high education group (Figure 6 and Annex Table 3).

## 4. Discussion

This article, based on data from the EHIS 3 study, shows that in the 2018 – 2020 survey period, the prevalence in Germany was slightly higher for diabetes and cardiovascular diseases (relative difference 14 % and 19 % respectively) and moderately higher for chronic respiratory diseases (44 %) compared to the European average of 29 countries. The prevalence patterns stratified by gender, age and education groups were similar in Germany and the European average.

The proportion of people with diabetes and chronic respiratory diseases with a self-assessed general health rating of very good or good was slightly higher in Germany (17 % and 7 % respectively) and moderately higher for cardiovascular diseases (34 %) than the average of the 29 European countries. At the same time, people with diabetes and chronic respiratory diseases (8 % and 12 % respectively), but not people with cardiovascular diseases, had slightly higher proportions of health-related limitations in everyday activities in Germany. The differences by gender, age and education, which are clearly pronounced in the European average for self-assessed general health and limitations in everyday activities, were only partially evident in Germany.

### 4.1 Diabetes mellitus

Based on the EHIS 3 data, the age-standardised 12-month prevalence of self-reported diabetes (European standard population 2013) was statistically significantly higher in Germany than in the average of the 29 European countries. However, the magnitude of the difference in prevalence can be considered as rather small, similar to the EHIS 2 data basis in 2014/2015 [1]. This is particularly true in comparison to the difference observed in 2014/2015 for other diseases such as high blood pressure, osteoarthritis and allergies [1]. Factors that can increase the prevalence of known diabetes include a less favorable risk factor constellation, but also improved early detection of diabetes and adequate diabetes care, which reduces the premature death of people affected by diabetes. The familiar pattern already described in a previous analysis of data from 2019/2020 for Germany, with higher prevalence in men than in women and increasing prevalence in both sexes with increasing age and decreasing education [18], can also be observed in a similar way for the European average.

According to the present analysis, significantly fewer adults in Germany who have diabetes rate their general health as very good or good (around one third) than was observed in the general population in an earlier study (around two thirds) [18], which confirms the corresponding observation in the European SHARE study [19]. While these differences are particularly pronounced in the lower age groups (18 – 64 years), they decrease in the upper age groups, which could possibly be related to the increasing burden of disease with increasing age in the general population. Compared to the European average, the proportion of people with diabetes in Germany with self-assessed very good or good health is higher. No significant differences can be observed here for the lower age range, but in the upper age groups, the proportion of people with diabetes in Germany with a self-assessed health rating of very good or good is significantly higher than the average for European countries.

More adults with diabetes in Germany report severe to moderate health-related limitations in everyday activities for at least six months (just under two-thirds) than in the general population in a previous study (just under half) according to this evaluation [18], with the difference again being greatest in the lower age groups (18 – 64 years). Compared to the European average, the proportion of people with diabetes in Germany is slightly higher, which is due in particular to the difference in the lower age range.

In order to interpret the results of self-assessed health and health-related everyday limitations, the various dimensions of these indicators, i.e. physical, social and emotional functional abilities and aspects relevant to illness [7], or the maintenance of independence and the ability to participate in various areas of everyday life such as housework, work, leisure activities [20] must be taken into account. For example, in the European SHARE study, depression, pain and self-assessed memory performance were most strongly associated with self-rated health in people with diabetes; however, the number of chronic comorbidities, polypharmacy, hospitalisation, vision and the current work situation were also identified as determinants [19]. However, the present results do not clarify the reasons for the disproportionately unfavorable proportions with regard to self-assessed health status and health-related limitations in everyday activities of people with diabetes of working age in Germany compared to the general population in Germany and with regard to everyday limitations also compared to the European average. This also applies to the observed less favorable proportions of self-assessed health and limitations in everyday activities in women compared to men and to the partially observed differences in education in Germany and on average in the European countries.

### 4.2 Cardiovascular diseases

The age-standardised 12-month prevalence of cardiovascular disease, which can be derived from the EHIS indicators, was higher in Germany than the average of the 29 European countries. It is important to note that the definition of cardiovascular disease derived from the EHIS indicators does not include all cardiovascular diseases, but does include self-reported chronic consequences as a result of a heart attack or stroke. This means that the self-assessed perception of the participants can also influence the prevalence, which can vary from one sociocultural background to another. In addition, a comparison with international analyses based on the ICD group of cardiovascular diseases, such as the Burden of Disease Study [21] or analyses of cardiovascular mortality [22] are not possible here.

As with diabetes, the magnitude of the difference between Germany and the average of the 29 European countries can be classified as comparatively small. Since higher prevalences are particularly evident in women under 65 and men between 45 and 74 years, a higher degree of diagnosis in Germany compared to the European average could also play a role. The observed prevalence pattern with higher prevalence in men than in women, increasing prevalence with increasing age and increasing prevalence with decreasing education and a stronger educational gradient in women than in men does not differ fundamentally between Germany and the European average. This is known and was also shown in analyses of cardiovascular indicators in the 2019/2020 and 2014/2015 EHIS data for Germany [18, 23, 24].

People with cardiovascular disease in the past twelve months in Germany rated their general state of health as very good or good more often than the European average, and this difference is most pronounced for cardiovascular disease when comparing the three disease groups considered. This difference was particularly attributable to older women and men over the age of 75. With regard to the self-assessed state of health, the dependencies on gender and education that can be observed in Germany and in European countries also appear to be relevant with regard to the care of patients. Women and people in the low and middle education groups were less likely to rate their general health as very good or good compared to men and people in the high education group. When interpreting these results, in addition to the different dimensions of self-rated health (see 4.1), the range of factors that influence the self-rated state of health must also be taken into account [25]. On the basis of the present study, however, no statements can be made about possible causes for the differences observed between Germany and the other European countries. Since, in addition to psychosocial and behavioral factors, material factors also contribute to social differences in self-rated health, ratio-based factors could contribute to differences in self-rated health at both national and international level [26, 27].

The prevalence of severe or moderate health-related limitations in everyday activities for at least six months among people with cardiovascular diseases was similar to the European average in Germany, unlike for diabetes and chronic respiratory diseases. As with self-assessed health, the clear differences by gender in Germany and in the European countries are striking, with higher proportions for women than for men; with regard to education, only the European average showed a clear trend in favor of decreasing education.

### 4.3 Chronic respiratory diseases

In the current EHIS 3 survey, the age-standardised 12-month prevalence of chronic respiratory diseases in Germany was higher than the average of the 29 European countries, with the difference in prevalence being more pronounced than for diabetes and cardiovascular diseases. Due to different indicator selection and methodology, no direct comparison with results of an analysis of the previous EHIS 2 survey is possible [1]. However, it was observed that the prevalence of chronic obstructive pulmonary disease (COPD) in Germany in 2014/2015 was above average in a European comparison [1]. For bronchial asthma, however, the prevalence estimates for both genders were at the average European level at that time. Similarly, it is reasonable to assume that the currently observed differences in the prevalence of chronic respiratory diseases between Germany and the European average are primarily due to differences in the prevalence of COPD. The results are consistent with reports of high hospital admission estimates for chronic lower respiratory diseases in Germany compared by the Organization for Economic Cooperation and Development [4]. The prevalence of chronic respiratory diseases in the present analysis, which is above the European average, particularly among women in Germany, is also consistent with the development of COPD prevalence based on the GBD data 2022 [28]. Compared to the beginning of the millennium, the prevalence of COPD in women in Germany increased by around 17 % by 2019. Germany thus recorded the second highest increase among the 28 EU countries included [28]. Similarly, the EHIS 3 2019/2020 showed an increase in COPD prevalence in women compared to the previous EHIS 2 survey 2014/2015, which was attributable to the 45 to 64 years age group. The observed increase may be related, among other things, to changes in smoking behavior over time [29]. The prevalence patterns observed in the current study in connection with age and education are also known. They have already been shown in previous analyses of EHIS data [18, 30, 31] and were also observed across Europe on the basis of the present study.

People with chronic respiratory diseases in the past twelve months in Germany rated their health as very good or good more often than the European average. This difference was particularly noticeable among women and men over the age of 65. The prevalence of severe or moderate health-related limitations in everyday activities for at least six months was higher among people with chronic respiratory diseases in Germany than the European average. This difference is particularly noticeable in the under 65 age group. On the basis of the present study, no conclusions can be drawn about possible causes for the observed differences in self-assessed health and health-related limitations in everyday activities between Germany and the other European countries among people with chronic respiratory diseases (see 4.1 and 4.2). In addition to differences in the type and severity of the chronic respiratory diseases under consideration and treatment adherence, the type and extent of co-morbidity or multimorbidity can also contribute to restrictions in the activity and quality of life of those affected and explain differences between the European countries and between the educational groups within Germany [32, 33].

### 4.4 Strengths and limitations

The strength of the EHIS lies above all in its ability to achieve a high degree of comparability between the participating European countries on a harmonized basis in terms of study design and data collection. This data can be analyzed with regard to possible differences, e.g. demographic or socio-economic inequalities. The EHIS thus represents an important information basis for European health reporting and policy [9].

Nevertheless, there are differences between the 29 countries with regard to the survey mode and wording of the questions, which could influence the results and thus also the comparisons between the countries [34, 35]. In addition, EHIS is based on information from surveys, which can lead to possible biases and differences compared to information from other data sources, such as examination surveys, medical findings/interviews or routine data (e.g. health insurance data). It should also be noted that survey data can also be distorted due to reporting errors or gaps in memory. In some countries, the survey is also based partly on information provided by relatives instead of the person being interviewed, which can potentially lead to distortions compared to information provided by the person themselves.

The comparison group of 29 European countries also includes Germany, meaning that differences between Germany and the European average tend to be underestimated. In the significance tests for gender-, age- and education-specific differences, it should be noted that the statistical power for the European average is higher compared to Germany due to the significantly larger sample size, i.e. even if the prevalence differences in Germany and the European average are comparable, for example, a significant difference is more likely to be found in the European average than in Germany, if a difference actually exists. With regard to the educational differences presented, no absolute or relative measures of inequality were calculated that take into account, for example, the overall distribution and the size of the educational groups (e.g. Slope Index and Relative Index of Inequality [36]), meaning that the extent of these educational differences cannot be directly compared between Germany and Europe on the basis of the results presented. Conclusions on differences in the extent of such health inequalities may vary depending on whether absolute and/or relative inequalities are considered [37, 38]. Furthermore, this article is limited to the presentation of prevalences and their differences in comparison between Germany and the European average. More in-depth considerations of differences between Germany and European country groups would be useful and necessary in order to better understand the differences observed here and to gain further insights.

### 4.5 Conclusion

This article shows that the prevalence of the three public health-relevant groups of non-communicable diseases diabetes, cardiovascular diseases and chronic respiratory diseases in Germany, derived from self-reported data, is slightly to moderately higher than the average of the 29 European countries considered. Despite the higher prevalence of these diseases and the partly higher proportion of self-reported health-related limitations in everyday activities in the presence of these diseases, adults with these diseases in Germany rate their health as better than the European average. This discrepancy cannot be adequately explained with the available data. However, various factors that have been investigated in previous studies could contribute to possible explanatory patterns. Compared to other European countries, Germany has a very well-developed healthcare system with extensive medical services and care offerings, e.g. with regard to medical aids, aftercare or support. Despite relatively high health-related limitations in everyday activities, this could lead to more confidence and fewer worries or fears in dealing with the illness and thus possibly contribute to better self-assessed general health. Cultural factors could also be relevant, as the perception of health and illness as well as dealing with limitations in everyday activities can be culturally different [39, 40]. Furthermore, from a methodological point of view, it could play a role that in some countries there is a greater stigma towards various health problems, which could influence the response behavior of the participants to the question of self-assessed general health.

In Germany, the differences in age and education were similar for chronic respiratory diseases, but less pronounced than the European average for diabetes and cardiovascular diseases. These differences and their causes must be investigated further, including differentiation of the various dimensions of self-assessed health and health-related everyday limitations, in order to derive concrete recommendations for action. Regular comprehensive information on health indicators is necessary in order to further develop strategies to improve the health situation and health equity. In particular, the finding of pronounced social inequality in the prevalence of chronic diseases to the detriment of lower education groups across Europe points to considerable potential for improvement.

Overall, the results underline the need for targeted health promotion, prevention strategies and care approaches that take into account not only behavioral but also more relation-context-based measures in order to reach all social groups and thus reduce health inequalities.

## Appendix

**Annex Table 1:** Age-standardised prevalence of diabetes mellitus, cardiovascular and chronic respiratory diseases in the past twelve months in Germany and Europe by gender, age group and education group (162,395 women, 139,565 men). Source: EHIS wave 3 (2018 – 2020)

	Diabetes				Cardiovascular diseases				Chronic respiratory diseases			
	Germany		Europe		Germany		Europe		Germany		Europe	
	%	(95 % CI)	%	(95 % CI)	%	(95 % CI)	%	(95 % CI)	%	(95 % CI)	%	(95 % CI)
**Women (total)**	**7.3**	**(6.6 – 8.0)**	**6.7**	**(6.5 – 6.9)**	**5.6**	**(5.0 – 6.2)**	**4.8**	**(4.7 – 5.0)**	**12.4**	**(11.5 – 13.4)**	**8.5**	**(8.2 – 8.7)**
Age group^*^												
18 – 44 years	2.0	(1.3 – 3.0)	1.3	(1.1 – 1.5)	1.3	(0.8 – 2.2)	0.6	(0.5 – 0.8)	10.1	(8.6 – 11 .8)	6.2	(5.8 – 6.6)
45 – 64 years	7.0	(5.9 – 8.3)	6.0	(5.6 – 6.3)	4.7	(3.8 – 5.8)	3.5	(3.2 – 3.7)	14.2	(12.7 – 15.9)	8.8	(8.4 – 9.2)
65 – 74 years	15.6	(13.2 – 18.2)	15.4	(14.7 – 16.1)	9.1	(7.5 – 11.1)	9.2	(8.6 – 9.7)	13.8	(11 .9 – 16.0)	11 .2	(10.6 – 11 .8)
75 years and older	18.7	(16.1 – 21.7)	19.6	(18.8 – 20.4)	20.4	(17.7 – 23.5)	19.8	(19.0 – 20.7)	14.2	(11 .9 – 16.8)	13.3	(12.6 – 14.1)
Education group												
Low	11 .2	(8.7 – 14.3)	9.4	(8.7 – 10.1)	9.5	(7.2 – 12.3)	7.2	(6.6 – 7.8)	16.3	(13.1 – 20.1)	10.9	(10.0 – 11.7)
Medium	7.1	(6.3 – 8.0)	5.7	(5.5 – 6.0)	5.0	(4.4 – 5.7)	4.2	(4.0 – 4.5)	12.0	(10.9 – 13.3)	8.0	(7.6 – 8.3)
High	4.4	(3.8 – 5.0)	4.1	(3.8 – 4.5)	3.6	(3.1 – 4.2)	3.1	(2.8 – 3.3)	10.4	(9.3 – 11.6)	6.8	(6.5 – 7.2)
**Men (total)**	**9.4**	**(8.6 – 10.3)**	**8.2**	**(7.9 – 8.4)**	**8.0**	**(7.3 – 8.8)**	**6.6**	**(6.4 – 6.9)**	**10.5**	**(9.6 – 11.4)**	**7.3**	**(7.1 – 7.6)**
Age group^*^												
18 – 44 years	2.3	(1.5 – 3.4)	1.3	(1.1 – 1.5)	0.7	(0.4 – 1.3)	0.6	(0.5 – 0.7)	7.5	(6.3 – 8.8)	5.0	(4.7 – 5.4)
45 – 64 years	10.8	(9.3 – 12.5)	8.5	(8.1 – 9.0)	8.2	(6.8 – 9.7)	5.7	(5.3 – 6.1)	11.5	(9.9 – 13.3)	7.0	(6.6 – 7.5)
65 – 74 years	20.0	(17.3 – 22.9)	18.9	(18.1 – 19.7)	18.6	(15.9 – 21.5)	15.0	(14.2 – 15.8)	14.8	(12.2 – 17.8)	10.5	(9.8 – 11 .3)
75 years and older	21.3	(18.3 – 24.7)	21.2	(20.2 – 22.2)	24.2	(20.8 – 27.9)	23.1	(22.0 – 24.1)	14.1	(11.5 – 17.2)	13.2	(12.4 – 14.1)
Education group												
Low	11 .8	(8.4 – 16.3)	10.3	(9.4 – 11.3)	9.5	(6.5 – 13.6)	8.4	(7.6 – 9.3)	12.3	(8.9 – 16.8)	9.3	(8.4 – 10.3)
Medium	10.3	(9.2 – 11 .6)	7.9	(7.5 – 8.3)	8.6	(7.6 – 9.8)	6.4	(6.1 – 6.7)	11.3	(10.1 – 12.7)	6.9	(6.5 – 7.3)
High	6.7	(6.1 – 7.4)	5.9	(5.5 – 6.2)	6.2	(5.6 – 7.0)	5.1	(4.8 – 5.5)	8.3	(7.4 – 9.3)	5.8	(5.5 – 6.1)
**Total**	**8.4**	**(7.8 – 8.9)**	**7.4**	**(7.3 – 7.6)**	**6.8**	**(6.3 – 7.3)**	**5.7**	**(5.6 – 5.9)**	**11.4**	**(10.8 – 12.1)**	**7.9**	**(7.7 – 8.1)**

^*^ Iceland and Malta: age 20 years and older

Europe: 29 European countries, including Germany

**Annex Table 2:** Age-standardised prevalence of self-assessed very good or good health status among people with diabetes mellitus, cardiovascular and chronic respiratory diseases in the past twelve months in Germany and Europe by gender, age group and education group (162,395 women, 139,565 men). Source: EHIS wave 3 (2018 – 2020)

	Diabetes				Cardiovascular diseases				Chronic respiratory diseases			
	Germany		Europe		Germany		Europe		Germany		Europe	
	%	(95 % CI)	%	(95 % CI)	%	(95 % CI)	%	(95 % CI)	%	(95 % CI)	%	(95 % CI)
**Women (total)**	**31.7**	**(27.3 – 36.4)**	**26.1**	**(24.7 – 27.5)**	**21.8**	**(17.6 – 26.7)**	**15.6**	**(14.2 – 17.1)**	**43.8**	**(39.8 – 47.8)**	**40.6**	**(39.0 – 42.1)**
Age group^*^												
18 – 44 years	49.5	(30.6 – 68.6)	51.1	(43.3 – 58.8)	48.6	(26.0 – 71.9)	41.9	(30.4 – 54.3)	64.5	(55.6 – 72.4)	65.8	(62.2 – 69.2)
45 – 64 years	23.6	(17.8 – 30.5)	27.6	(25.3 – 30.2)	19.3	(13.6 – 26.8)	19.0	(16.5 – 21.9)	35.5	(30.3 – 41.0)	36.7	(34.4 – 39.0)
65 – 74 years	32.1	(24.7 – 40.5)	24.0	(21.7 – 26.3)	17.3	(11 .0 – 26.2)	13.6	(11 .6 – 15.9)	35.0	(28.1 – 42.6)	26.6	(24.1 – 29.2)
75 years and older	32.8	(25.4 – 41.2)	20.0	(17.9 – 22.3)	19.1	(13.7 – 26.0)	11 .4	(9.7 – 13.3)	20.9	(15.0 – 28.3)	15.7	(13.7 – 17.9)
Education group												
Low	27.4	(17.9 – 39.4)	21.5	(18.5 – 25.0)	22.5	(12.3 – 37.6)	13.4	(9.8 – 18.2)	25.5	(16.8 – 36.7)	26.1	(22.6 – 30.0)
Medium	31.7	(26.4 – 37.6)	30.0	(27.5 – 32.6)	19.9	(15.5 – 25.1)	17.1	(15.1 – 19.4)	45.0	(39.8 – 50.3)	43.2	(40.9 – 45.6)
High	41.5	(34.6 – 48.8)	38.0	(34.2 – 41.9)	30.8	(23.7 – 39.1)	23.2	(19.9 – 26.9)	61.7	(56.3 – 66.8)	55.6	(52.7 – 58.5)
**Men (total)**	**38.9**	**(34.4 – 43.5)**	**34.4**	**(32.9 – 35.9)**	**27.7**	**(23.6 – 32.2)**	**21.2**	**(19.8 – 22.7)**	**45.8**	**(41.3 – 50.4)**	**43.4**	**(41.6 – 45.2)**
Age group^*^												
18 – 44 years	33.3	(17.9 – 53.3)	45.6	(37.0 – 54.4)	30.0	(11.2 – 59.3)	37.0	(28.2 – 46.8)	74.2	(65.8 – 81.1)	74.1	(70.9 – 77.1)
45 – 64 years	36.0	(28.9 – 43.8)	36.6	(34.0 – 39.2)	30.5	(22.7 – 39.6)	24.3	(21.3 – 27.6)	39.6	(32.3 – 47.4)	40.6	(37.4 – 43.8)
65 – 74 years	41.7	(34.2 – 49.6)	34.5	(32.1 – 37.0)	27.3	(21.1 – 34.7)	21.5	(19.3 – 23.9)	30.1	(22.5 – 38.9)	27.3	(24.4 – 30.5)
75 years and older	42.5	(34.3 – 51.1)	29.0	(26.4 – 31.7)	24.9	(18.5 – 32.6)	17.2	(15.1 – 19.4)	21.4	(15.2 – 29.3)	17.1	(14.9 – 19.4)
Education group												
Low	36.4	(21.5 – 54.3)	29.3	(24.7 – 34.2)	21.2	(9.0 – 42.2)	15.2	(11 .1 – 20.5)	32.0	(18.8 – 48.9)	30.8	(26.0 – 36.2)
Medium	35.0	(29.4 – 41.1)	34.7	(32.3 – 37.3)	24.5	(19.2 – 30.8)	21.5	(19.2 – 23.9)	42.9	(37.0 – 49.1)	45.7	(42.9 – 48.5)
High	51.0	(46.1 – 55.8)	45.1	(42.2 – 48.0)	40.7	(35.1 – 46.5)	34.0	(30.9 – 37.2)	64.7	(59.3 – 69.7)	61.1	(58.1 – 64.0)
**Total**	**35.8**	**(32.6 – 39.1)**	**30.7**	**(29.6 – 31.7)**	**25.3**	**(22.3 – 28.6)**	**18.9**	**(17.8 – 19.9)**	**44.7**	**(41.7 – 47.8)**	**41.9**	**(40.7 – 43.0)**

^*^ Iceland and Malta: age 20 years and older

Europe: 29 European countries, including Germany

**Annex Table 3:** Age-standardised prevalence of self-assessed severe or moderate health-related limitations in everyday activities for at least six months in among people with diabetes mellitus, cardiovascular and chronic respiratory diseases in the past twelve months in Germany and Europe by gender, age group and education group (162,395 women, 139,565 men). Source: EHIS wave 3 (2018 – 2020)

	Diabetes				Cardiovascular diseases				Chronic respiratory diseases			
	Germany		Europe		Germany		Europe		Germany		Europe	
	%	(95 % CI)	%	(95 % CI)	%	(95 % CI)	%	(95 % CI)	%	(95 % CI)	%	(95 % CI)
**Women (total)**	**71.7**	**(67.2 – 75.7)**	**65.1**	**(63.6 – 66.5)**	**82.3**	**(77.8 – 86.0)**	**79.2**	**(77.8 – 80.5)**	**64.1**	**(60.1 – 67.9)**	**58.2**	**(56.7 – 59.7)**
Age group^*^												
18 – 44 years	79.0	(60.5 – 90.2)	47.8	(40.1 – 55.7)	75.4	(53.2 – 89.2)	64.5	(53.6 – 74.0)	47.6	(39.3 – 55.9)	37.1	(33.6 – 40.7)
45 – 64 years	75.0	(67.1 – 81.5)	60.5	(57.6 – 63.2)	89.4	(83.6 – 93.4)	75.2	(72.2 – 77.9)	70.6	(64.9 – 75.7)	60.5	(58.1 – 62.9)
65 – 74 years	63.4	(54.8 – 71.2)	61.1	(58.6 – 63.6)	72.4	(61.1 – 81.5)	73.0	(70.0 – 75.7)	69.9	(62.4 – 76.4)	66.9	(64.3 – 69.5)
75 years and older	73.0	(65.4 – 79.4)	77.2	(75.2 – 79.1)	84.4	(77.7 – 89.3)	86.5	(84.8 – 88.1)	84.4	(77.6 – 89.4)	83.0	(81.0 – 84.9)
Education group												
Low	77.4	(65.5 – 86.0)	70.9	(67.5 – 74.1)	88.4	(79.2 – 93.8)	83.9	(81.0 – 86.4)	77.0	(64.8 – 85.9)	68.8	(64.8 – 72.6)
Medium	69.1	(63.6 – 74.1)	59.9	(57.4 – 62.4)	81.8	(76.6 – 86.1)	76.2	(73.9 – 78.3)	61.6	(56.2 – 66.7)	54.7	(52.4 – 57.1)
High	66.7	(59.5 – 73.2)	51.9	(47.9 – 55.8)	76.3	(69.1 – 82.2)	70.4	(66.3 – 74.2)	51.0	(45.2 – 56.7)	45.5	(42.6 – 48.4)
**Men (total)**	**61.0**	**(56.3 – 65.5)**	**57.0**	**(55.5 – 58.5)**	**75.2**	**(70.6 – 79.3)**	**74.2**	**(72.7 – 75.6)**	**64.8**	**(60.4 – 69.0)**	**56.8**	**(55.1 – 58.5)**
Age group^*^												
18 – 44 years	50.0	(30.7 – 69.3)	44.8	(36.3 – 53.5)	66.2	(38.1 – 86.2)	63.0	(53.7 – 71.4)	42.1	(33.8 – 50.8)	33.5	(30.1 – 37.0)
45 – 64 years	65.2	(57.5 – 72.2)	53.1	(50.4 – 55.9)	75.0	(66.0 – 82.2)	70.1	(66.9 – 73.1)	71.0	(63.9 – 77.2)	57.7	(54.6 – 60.8)
65 – 74 years	60.0	(52.1 – 67.4)	54.6	(52.1 – 57.1)	76.8	(70.0 – 82.5)	71.7	(69.3 – 74.1)	77.8	(70.2 – 83.9)	68.8	(65.7 – 71.8)
75 years and older	60.6	(52.0 – 68.5)	67.0	(64.4 – 69.5)	74.9	(66.0 – 82.2)	80.0	(77.6 – 82.3)	81.5	(69.4 – 89.5)	78.6	(75.6 – 81.4)
Education group												
Low	61.6	(43.3 – 77.1)	62.3	(57.4 – 67.0)	80.9	(60.0 – 92.2)	79.9	(75.1 – 83.9)	88.6	(72.3 – 95.9)	71.5	(67.1 – 75.6)
Medium	63.3	(57.1 – 69.0)	56.1	(53.6 – 58.6)	76.3	(69.5 – 81.9)	72.4	(69.8 – 74.9)	65.6	(59.4 – 71.3)	54.6	(51.8 – 57.3)
High	54.4	(49.5 – 59.2)	48.9	(46.0 – 51.8)	67.2	(61.3 – 72.5)	65.5	(62.2 – 68.6)	47.6	(41.9 – 53.4)	42.0	(39.0 – 45.1)
**Total**	**65.6**	**(62.3 – 68.8)**	**60.6**	**(59.6 – 61.7)**	**78.1**	**(74.8 – 81.0)**	**76.3**	**(75.2 – 77.3)**	**64.5**	**(61.5 – 67.3)**	**57.6**	**(56.4 – 58.7)**

^*^ Iceland and Malta: age 20 years and older

Europe: 29 European countries, including Germany
